# The impact of spiritually-oriented therapy on the rehabilitation of mentally ill patients with a religious worldview

**DOI:** 10.1192/j.eurpsy.2025.2178

**Published:** 2025-08-26

**Authors:** A. Magay

**Affiliations:** Mental Health Research Centre, Moscow, Russian Federation

## Abstract

**Introduction:**

Spirituality and religiosity have a significant effect on mental health. Biopsychosocyospiritual model in psychiatry is used in multiple researches in Europe and Russia as well (Hefti 2013; 24(2) 119-129). Many patients use religious beliefs and actions to cope with mental disease (Tepper et al., 2001; *2*(5) 660-665). The pathological religiosity phenomenon determining the severity of mental disease is described (Borisova 2020; 120(1) 103-110). Spiritually-oriented therapy methods are aimed at pathological religiosity correction and assist in mental well-being achievement.

**Objectives:**

To assess the effect of spiritually-oriented therapy on mental health of religious patient during comprehensive rehabilitation.

**Methods:**

42 patients with endogenous mental schizophrenic and affective spectrum disorders who participated in comprehensive rehabilitation using spiritually-oriented therapy (26 women and 16 men) were examined. There were used psychometrical (SF-36, PIL, Schwartz Value Survey – SVS, B-RCOPE) and statistical methods.

**Results:**

In a therapeutic environment in the religious community, 42 patients received psychopharmacological treatment and participated in multimodal rehabilitation throughout the year. Psychoeducational, art-therapeutic, spiritually-oriented modules were used in rehabilitation. 13 (31%) ceased the rehabilitation early, 29 (15 women and 14 men) underwent the entire rehabilitation. 73% of them had a pronounced improvement in quality of life indicators, 84% had a meaningfulness of life increase, a transformation of value system with the dominance of “security,” “kindness” and “traditions”, a higher cohesion of values. Rehabilitated patients had a more harmonious religiosity structure with a predominance of inner religiosity, they used religious behavior to cope with the disease which correlated with a decrease in the intensity of pathological religiosity manifestations.

**Conclusions:**

The use of spiritually-oriented therapy methods in the rehabilitation of mentally ill patients with a religious worldview promote to correct the manifestations of pathological religiosity and to cope with mental disease. Patients have a qualitative and quantitative change in value system and religious beliefs, mastering adaptive styles of religious behavior. Spiritually-oriented therapy methods are not an alternative to natural religious life, but complement it, taking into account the existing mental disorders.

**Disclosure of Interest:**

None Declared

